# Topographic distinction in long-term value signals between presumed dopamine neurons and presumed striatal projection neurons in behaving monkeys

**DOI:** 10.1038/s41598-020-65914-0

**Published:** 2020-06-02

**Authors:** Kazuki Enomoto, Naoyuki Matsumoto, Hitoshi Inokawa, Minoru Kimura, Hiroshi Yamada

**Affiliations:** 1 Department of Physiology, Kyoto Prefectural University of Medicine, Kyoto, 602-8566 Japan; 2 Center for Information and Neural Networks, National Institute of Information and Communications Technology, Osaka, 565-0871 Japan; 3 Brain Science Institute, Tamagawa University, Machida, Tokyo 194-8610 Japan; 4 Division of Food and Health Sciences, Faculty of Environmental and Symbiotic Sciences, Prefectural University of Kumamoto, Kumamoto, 862-8502 Japan; 5 Graduate School of Comprehensive Human Sciences, University of Tsukuba, 1-1-1 Tenno-dai, Tsukuba, Ibaraki 305-8577 Japan; 6 Transborder Medical Research Center, University of Tsukuba, 1-1-1 Tenno-dai, Tsukuba, Ibaraki 305-8577 Japan; 7 Division of Biomedical Science, Faculty of Medicine, University of Tsukuba, 1-1-1 Tenno-dai, Tsukuba, Ibaraki 305-8577 Japan

**Keywords:** Neuroscience, Reward

## Abstract

Nigrostriatal dopamine (DA) projections are anatomically organized along the dorsolateral-ventromedial axis, conveying long-term value signals to the striatum for shaping actions toward multiple future rewards. The present study examines whether the topographic organization of long-term value signals are observed upon activity of presumed DA neurons and presumed striatal projection neurons (phasically active neurons, PANs), as predicted based on anatomical literature. Our results indicate that DA neurons in the dorsolateral midbrain encode long-term value signals on a short timescale, while ventromedial midbrain DA neurons encode such signals on a relatively longer timescale. Activity of the PANs in the dorsal striatum is more heterogeneous for encoding long-term values, although significant differences in long-term value signals were observed between the caudate nucleus and putamen. These findings suggest that topographic DA signals for long-term values are not simply transferred to striatal neurons, possibly due to the contribution of other projections to the striatum.

## Introduction

Animals maximize their benefits by assessing future events on a timescale that incorporates a series of actions and their multiple rewards. Previous findings suggest that not only the values of immediate rewards, but also long-term values of multiple future rewards (i.e. those associated with multiple future rewards through a series of actions) are learned based on signals from midbrain dopamine (DA) neurons, which convey reward values to cortical and sub-cortical structures^1–5^. However, it remains unclear how DA value signals are reflected in neuronal activity within these target brain regions.

Previous studies have reported that DA neurons in the midbrain exhibit functional heterogeneity and topography^6–10^, often with a dorsolateral-ventromedial arrangement^11^. For example, DA neurons in the dorsolateral and ventromedial midbrain encode motivational salience and motivational value, respectively^12^. Furthermore, DA neurons in the ventromedial midbrain exhibit greater response sensitivity to reward values than those in the dorsolateral midbrain^13^. Novelty signals reflected on the DA terminal are also different between dorsolateral and ventromedial DA projections^14^. These studies indicate that midbrain DA neurons may also exhibit topographic differences in the encoding of long-term values, and that topographic DA signals along the dorsolateral-ventromedial axis may shape topographic differences in value signals represented within the target brain regions.

The striatum is one of the brain regions under predominant control of the DA signal, receiving dense DA projections via the nigrostriatal pathway and exhibiting topographic organization^15,16^. The striatum is thought to learn and perform reward-directed actions^17–20^. Striatal output neurons, also known as phasically active neurons (PANs), alter their activation properties via dopamine D1- and D2-like receptors^21–23^ while they exhibit value-dependent activity during eye and skeletomotor movements^24–30^. Indeed, similar to DA neurons, PANs represent the long-term values of multiple future rewards^31^, suggesting a predominant influence of DA signals on the activity of striatal neurons. However, little is known regarding the mechanism by which value signals carried by DA neurons are represented by activity of PANs in the striatum. This is partly because no studies have directly compared long-term value signals between DA neurons and PANs using a single behavioral task.

In the present study, we specifically examine the heterogeneity of long-term value signals in terms of topography by analyzing the recording locations of neurons in our datasets, in which we have already demonstrated that DA neurons and PANs encode long-term values using the same behavioral task^5,31^. Based on nigrostriatal projections, we examined (1) whether the long-term value signals of DA neurons exhibit a topography along the dorsolateral-ventromedial axis; (2) whether PANs also exhibit topographic differences in the encoding of long-term values; (3) whether the topographical organization patterns of DA neurons and PANs reflect that predicted by anatomical literature^15,16^. Our results indicate that both DA neurons and PANs exhibit topographic differences in the encoding of long-term values. However, their topographic characteristics did not completely align with the nigrostriatal anatomical projections, suggesting that additional contributions from other networks are likely involved.

## Results

### Previous findings: behavioral and neuronal representation of long-term values during a multi-step choice task

**Figure 1:**
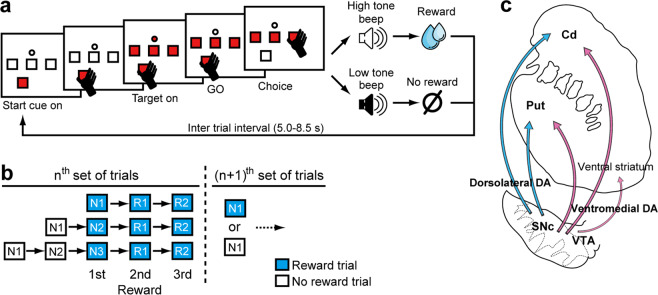
Multi-step choice task. **(a)** Sequence of events in a single trial. Monkeys chose one of three targets in a trial to find one rewarding target. Following the ITI, monkeys chose a target again based on the reward and no-reward outcomes in the previous trials. **(b)** Schematic drawing of a series of choices to obtain multiple rewards. After finding a rewarding target, monkeys were required to repeat the rewarded choices in the first and second repeat trials (R1 and R2). Monkeys obtained two (monkey RO, no R2 trials) or three rewards (monkeys SK, CC, and TN) in a series of trials. **(c)** Schematic drawing of the nigrostriatal projection. Anatomical projections from the midbrain to the striatum based on Haber *et al*., 2000 are shown.

Two prior studies conducted in our laboratory have shown that DA neurons and PANs encode the expected values of multiple future rewards during a series of choices^5,31^. In these studies, monkeys performed the same behavioral task (see Materials and Methods, multi-step choice task, Fig. 1a,b), in which they first searched for and found a rewarding target from among three alternatives on a trial-and-error basis (Fig. 1b; N1, N2, and N3 trial types), following which they earned additional rewards by choosing the rewarding target in subsequent trials (Fig. 1b; R1 and R2 trial types). The monkeys’ task performances were also previously examined. After significant training for approximately six to ten months, the monkeys efficiently performed the multi-step choice task (Supplementary Table 1), with more than 70% of rewarded choice in N3 trials. The percentages of monkeys who successfully found a rewarding target (i.e., reward probability) progressively increased along the first (N1, 17–33%), second (N2, 47–50%), and third choices (N3, 76–89%) during search trials. The percentage to find a rewarding target in N3 trials was not 100% because the monkeys sometimes chose one of the non-rewarded targets as all three target options were presented. However, once they found the rewarding target, the reward probability surpassed 90% in the first (R1, 93–97%) and second (R2, 95–97%) repeat trials.

In the multi-step choice task, monkeys expect to receive multiple rewards through a series of choices after completing the training. We modeled the expectation of multiple future rewards through a series of choices (i.e. long-term value) using a standard reinforcement learning paradigm^5,31^ (see Analysis of Behavioral Data section in Materials and Methods). Briefly, the estimated long-term value, *V(S*_*t*_), represents the summation of expected multiple future rewards (Supplementary Fig. 1a). The value of future rewards in this estimation is discounted by the number of steps required to obtain the rewards using the discount factor, *γ*, which represents the timescale of the expectation. Larger values of *γ* reflect longer timescales for the estimated long-term value, which yield an inverse-V shape of the long-term values through a series of choices. If *γ* is zero, no future rewards are expected in this estimation, meaning that the reward value is estimated based on immediate rewards in the current trial (probability × magnitude). We previously demonstrated that anticipatory licking behavior in monkeys is not well explained by expectations of a single upcoming reward in an ongoing trial (i.e. probability × magnitude in each trial type, *γ* equal to zero), but by the long-term value (Supplementary Fig. 1b,c). The inverse-V shape of the long-term value best explains the normalized average durations of licking (*γ* = 0.66, Supplementary Fig. 1c). In the two earlier studies, we also revealed that estimated long-term values are encoded by the activity of DA neurons^5^ and PANs^31^.

**Figure 2:**
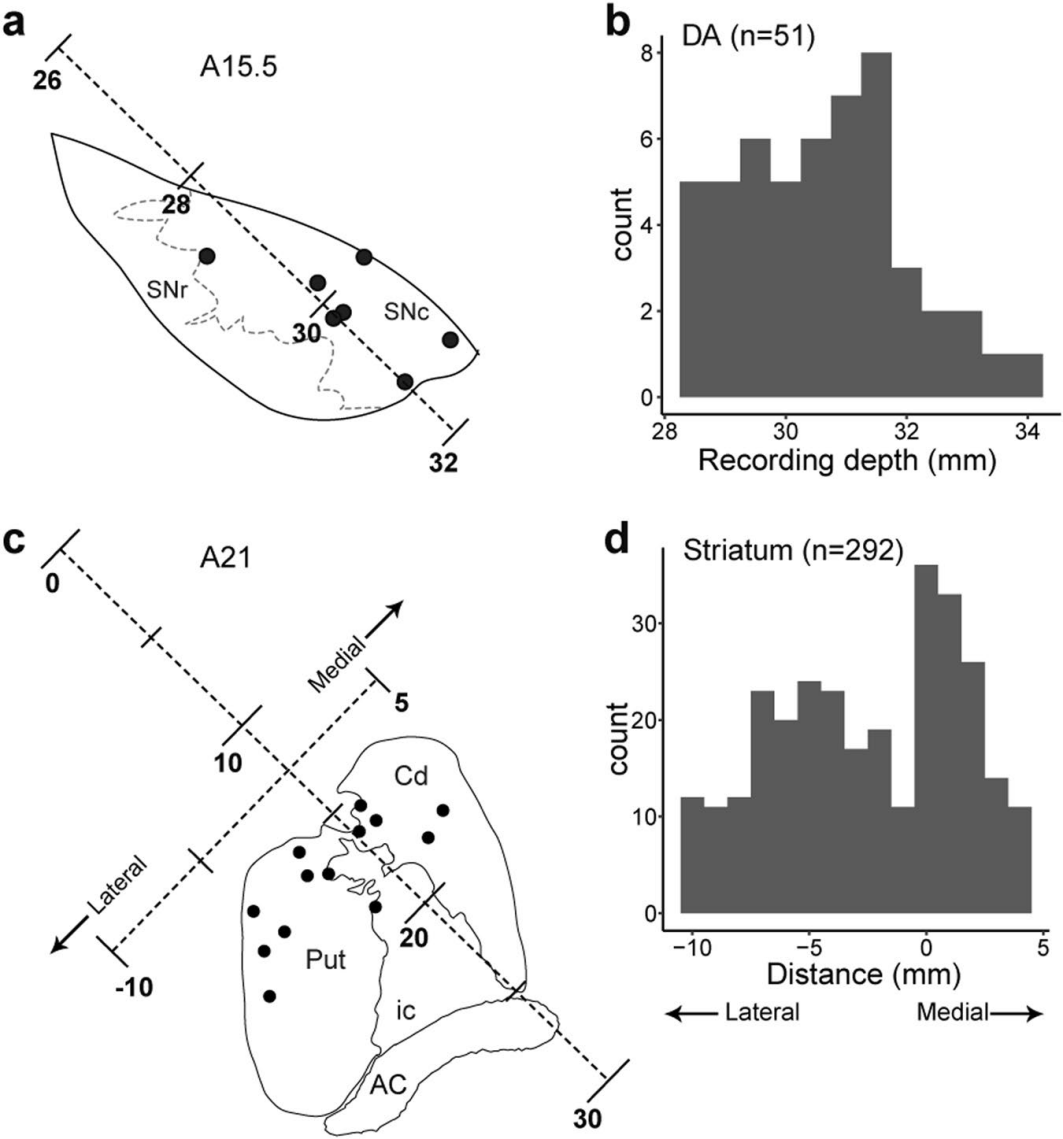
Recording locations of DA neurons and PANs. **(a)** Recording sites of DA neurons (black dots) in a representative coronal section reconstructed histologically at 15.5 mm anterior (A15.5). Depth from the cortical surface was represented along the electrode insertion. SNr, substantia nigra pars reticulata; SNc, substantia nigra pars compacta. **(b)** Numbers of DA neurons at each recording depth. **(c)** Recording sites of PANs in a representative coronal section reconstructed histologically at 21 mm anterior (A21). Depth from the cortical surface was represented along the electrode insertion. Mediolateral location relative to the edge of the caudate nucleus was represented along the axis orthogonal to the electrode insertion. Cd, caudate nucleus; Put, putamen; ic, internal capsule; AC, anterior commissure. **(d)** Numbers of PANs at each mediolateral location. See Supplementary Fig. 2 for more detail.

In the current study, we reanalyzed the long-term value signals by specifically focusing on topographic differences (i.e. recording location) that were not examined previously in our dataset. As per anatomical literature (Fig. 1c), we investigated whether dorsolateral and ventromedial midbrain DA neurons exhibit differences in long-term value coding by comparing recording depth from the cortical surface (Fig. 2a,b and Supplementary Fig. 2a), a procedure used in the previous monkey study^12^. For striatal neurons, we investigated whether PANs in the caudate nucleus and putamen exhibit differences in long-term value coding by examining the mediolateral recording axis via histological reconstruction (Fig. 2c,d). We also examined anterior-posterior and dorsoventral differences throughout the dorsal striatum (Supplementary Fig. 2b, see Materials and Methods). We reanalyzed recordings from a total of 51 DA neurons and 292 PANs in two monkeys each (Supplementary Table 2). Note that most of the DA neurons were recorded from the substantia nigra par compacta (SNc), while the PANs were from whole dorsal striatum. Note also that we eliminated the data recorded from DA neurons during an early stage of learning in this analysis, as they exhibited learning-dependent changes in their firing patterns^5^, while all PAN recordings were made after the monkeys had learned the task^31^. The neurons analyzed in the present study were recorded from after monkeys learned the multi-step choice task, a threshold that was defined as when they achieved more than 80% of the highest stable rewarded rates in N3 trials in a week (100% in monkey SK, 84% in monkey CC, 100% in monkey RO, and 100% in monkey TN).

### Ventromedial DA neurons encode multiple future rewards on a longer timescale than dorsolateral DA neurons

**Figure 3:**
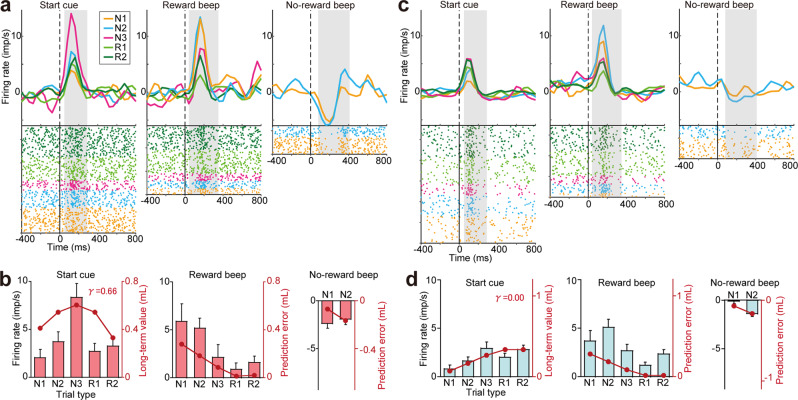
Representative activity of DA neurons in the ventromedial and dorsolateral parts of the midbrain. **(a)** Raster plots and histograms of DA neuron activity recorded from the ventromedial part of the midbrain after the start cue (left panel), reward beep (center panel), and no-reward beep (right panel). Firing rates relative to baseline activity are shown. Spikes were sorted according to trial as follows: N1 (orange), N2 (cyan), N3 (magenta), R1 (light green), and R2 (dark green). Hatched gray areas represent the time windows used to measure response amplitude in each histogram. (**b)** Response amplitude of the example neuron shown in **(a)** after the start cue, reward beep, and no-reward beep for each trial type (bar graph, mean and SE). Superimposed line plots indicate the long-term value (start cue) and the prediction errors of long-term values (reward and no-reward beeps) estimated based on the best-fit *γ* value (see Materials and Methods). A single best-fit *γ* value was estimated against responses to start cue, reward beep, and no-reward beep simultaneously. **(c,d)** Same as **(a,b)** but for a DA neuron recorded from the dorsolateral part of the midbrain.

Figure 3a shows example activity of a DA neuron located within the deeper, ventromedial part of the midbrain (Fig. 3a, depth = 33.0 mm). This DA neuron exhibited significant responses following illumination of the start cue, with an increase in firing rates. The neuron also showed increases in the firing rate after reward beeps, whereas the firing rate decreased after no-reward beeps. The magnitude of cue responses progressively increased from N1 to N3 during search trials and decreased in repeat trials (Fig. 3b, left, R1 and R2). The inverse-V-shaped response pattern was best explained by long-term values with a medium-range timescale (*γ* = 0.66). A similar pattern of responses was observed for another DA neuron present in the shallower, dorsolateral part of the midbrain (Fig. 3c, depth = 28.4), although activity modulation in this neuron was best explained by values of immediate rewards (*γ* = 0.00, Fig. 3d). These DA responses appeared to reflect regional differences in the long-term values of future rewards along the dorsolateral-ventromedial axis.

**Figure 4:**
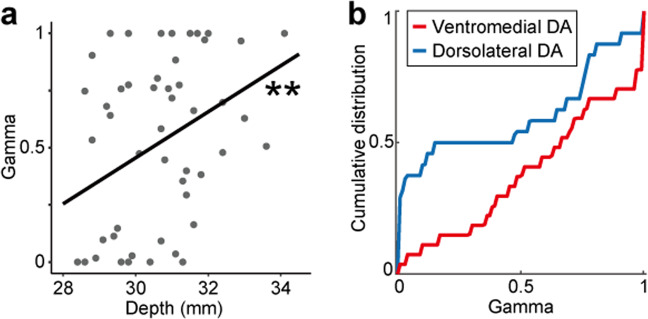
Ventromedial DA neurons encode multiple future rewards on a longer timescale than dorsolateral ones. **(a)** Scatter plot of the estimated *γ* value against recording depth in each DA neuron. Regression line is presented in black. Asterisks indicate the significance of the regression coefficient (**p < 0.01). **(b)** Cumulative distributions of the estimated *γ* values in dorsolateral (blue) and ventromedial (red) DA neurons.

To quantitatively examine how these activity differences along the dorsolateral-ventromedial axis reflect the timescale of long-term value coding, we fitted the standard reinforcement learning model used in our previous studies and estimated the *γ* value for each activity of DA neurons (see Materials and Methods). The estimated *γ* values for each DA neuron exhibited significant regional differences. Larger *γ* values were observed for deeper DA neurons as demonstrated by the positive regression coefficient of the recording depth (Fig. 4a, linear regression, regression coefficient, *r* = 0.101, *p* = 0.006, R^2^ = 0.15). If the DA data was divided into the dorsolateral and ventromedial DA neurons based on the anatomical criteria (see Materials and Methods), the cumulative distributions of *γ* differed significantly between the dorsolateral (n = 24) and ventromedial (n = 27) populations (Fig. 4b, Kolmogorov-Smirnov test, *p* = 0.031). Thus, these findings suggest that ventromedial DA neurons represent long-term reward values on a longer timescale than dorsolateral DA neurons. Note that we also examined changes in the firing rates of DA neurons without the reinforcement learning model (Supplementary Results and Supplementary Fig. 3). Moreover, the recording depth of the DA neurons did not affect the learning rate *α*, but affected only *γ* if we fit the reinforcement learning model to the data with two free parameters *γ* and *α* simultaneously (Supplementary Fig. 4, regression coefficient; *γ*, *r* = 0.101, *p* = 0.006, R^2^ = 0.143; *α*, *r* = 0.00161, *p* = 0.966, R^2^ = 0.0000376).

### Heterogeneous coding of long-term values by PANs in the caudate nucleus and putamen

**Figure 5:**
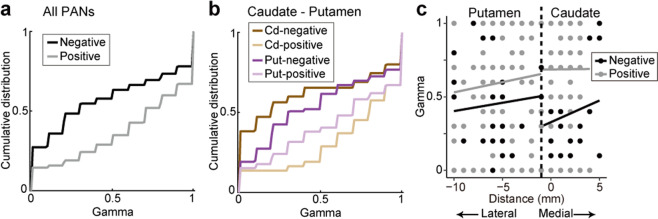
PANs in the caudate nucleus encode future rewards on shorter and longer timescales than PANs in the putamen. **(a)** Cumulative distributions of the estimated *γ* values in positive-coding (gray) and negative-coding type PANs (black). **(b)** Same as **(a)** but for PANs in the caudate nucleus (positive-coding type, yellow; negative-coding type, brown) and putamen (positive-coding type, light purple; negative-coding type, dark purple). **(c)** Scatter plot of the estimated *γ* value against mediolateral recording axis in the caudate nucleus and putamen. Regression lines are presented for positive- (gray) and negative-coding type PANs (black) in each of the caudate nucleus and putamen, respectively.

We next analyzed the recording locations of PANs, which encode long-term values. As reported previously, PANs exhibited increases in phasic activity during one or more behavioral events during the multi-step choice task, as well as positive or negative regression coefficients of the long-term reward value on several timescales (Figs. 2 and 3 in Yamada *et al*., 2013, shown again in Supplementary Fig. 5). We analyzed the 280 activities of PANs reported previously, which encode long-term values (0 ≤ *γ* ≤ 1) among 656 task-related activities shown by 292 PANs. Significant differences in the discount factor *γ* were observed between PANs with positive and negative coding types for long-term value signals (Fig. 5a, Kolmogorov-Smirnov test, *p* < 0.00001, see also Figs. 5 and 6 in Yamada *et al*., 2013). In the present study, we examined whether these long-term value signals differ based on recording location; caudate nucleus vs. putamen (i.e. mediolateral difference, Fig. 2c,d; ML -1 indicates the edge of the caudate nucleus), recording depth (dorsolateral-ventromedial axis), and anterior-posterior location (AP level) (Fig. 2c,d and Supplementary Fig. 2b).

**Figure 6:**
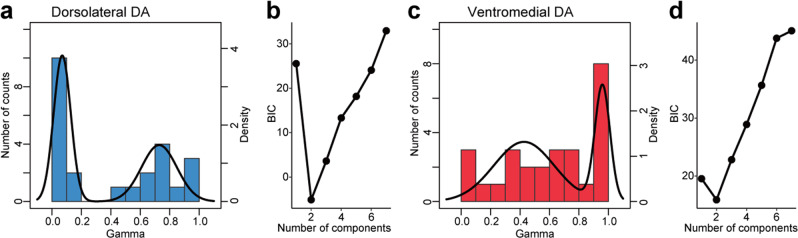
Long-term value signals encoded by DA neurons are composed of two subgroups. **(a)** Distribution of *γ* values for dorsolateral DA neurons (bar graph). The best-fitting model is indicated by the black line. **(b)** Plots of the estimated Bayesian information criterion (BIC) for the data in **(a)** for each model, which included one to seven components. **(c,d)** Same as **(a,b)** but for ventromedial DA neurons.

Our examination of recording locations revealed that caudate PANs exhibited significantly greater differences in the discount factor between positive and negative-coding types than those in the putamen (Fig. 5b); positive-coding type PANs preferred larger *γ* values (longer timescale, yellow), whereas negative-coding type PANs preferred smaller *γ* values (shorter timescale, brown) (Kolmogorov-Smirnov test, *p* < 0.00001). Although similar tendencies were observed for the discount factors of PANs in the putamen, the differences did not reach statistical significance (Fig. 5b, light and dark purple, Kolmogorov-Smirnov test, *p* = 0.0578). When we applied a regression analysis to the PAN data in each of the caudate nucleus and putamen, PANs did not show dependence on the mediolateral differences (Fig. 5c, linear regression, regression coefficient; caudate, mediolateral difference, *r* = 0.0169, *p* = 0.517, coding type, *r* = 0.316, *p* < 0.00001, R^2^ = 0.149; putamen, mediolateral difference, *r* = 0.0128, *p* = 0.292, coding type, *r* = 0.140, *p* = 0.0248, R^2^ = 0.0391). Note that the estimated *γ* values were not differentiated based on recording depth or AP level in either the caudate nucleus (multiple regression analysis, regression coefficient; recording depth, *r* = −0.0215, *p* = 0.325; AP level, *r* = 0.00534, *p* = 0.636, R^2^ = 0.00795) or putamen (recording depth, *r* = 0.0183, *p* = 0.366; AP level, *r* = 0.0137, *p* = 0.112, R^2^ = 0.0374). Thus, long-term value coding employed by PANs was similar, but not identical, between the caudate nucleus and putamen, even within the dorsal part of the striatum where both receive DA signals from the SNc.

### Long-term value signals encoded by DA neurons and PANs as a mixture of heterogeneous subgroups

Lastly, we examined differences in long-term value signals across DA neurons and PAN populations by examining how many subgroups existed in their distribution of long-term value signals. We fitted a mixture of Gaussian distribution models to the data in order to identify the number of components that best explain the distribution of *γ* values (See Materials and Methods). Bayesian information criteria (BIC) were estimated to define the best model, which exhibits the smallest BIC value amongst alternatives. For both dorsolateral and ventromedial DA neurons, a two-component model best explained the *γ* distribution (Fig. 6a–d). Dorsolateral DA neurons were composed of a subgroup of small (mean ± SD: 0.070 ± 0.058, n = 12 neurons) and medium-to-large *γ* values (mean ± SD: 0.73 ± 0.12, n = 12), in which the former represents values close to immediate rewards. In contrast, ventromedial DA neurons were composed of small-to-large (mean ± SD: 0.43 ± 0.20, n = 18) and large (mean ± SD: 0.96 ± 0.051, n = 9) *γ* values, with the latter representing an almost perfect prediction of multiple future rewards through a series of choices.

**Figure 7:**
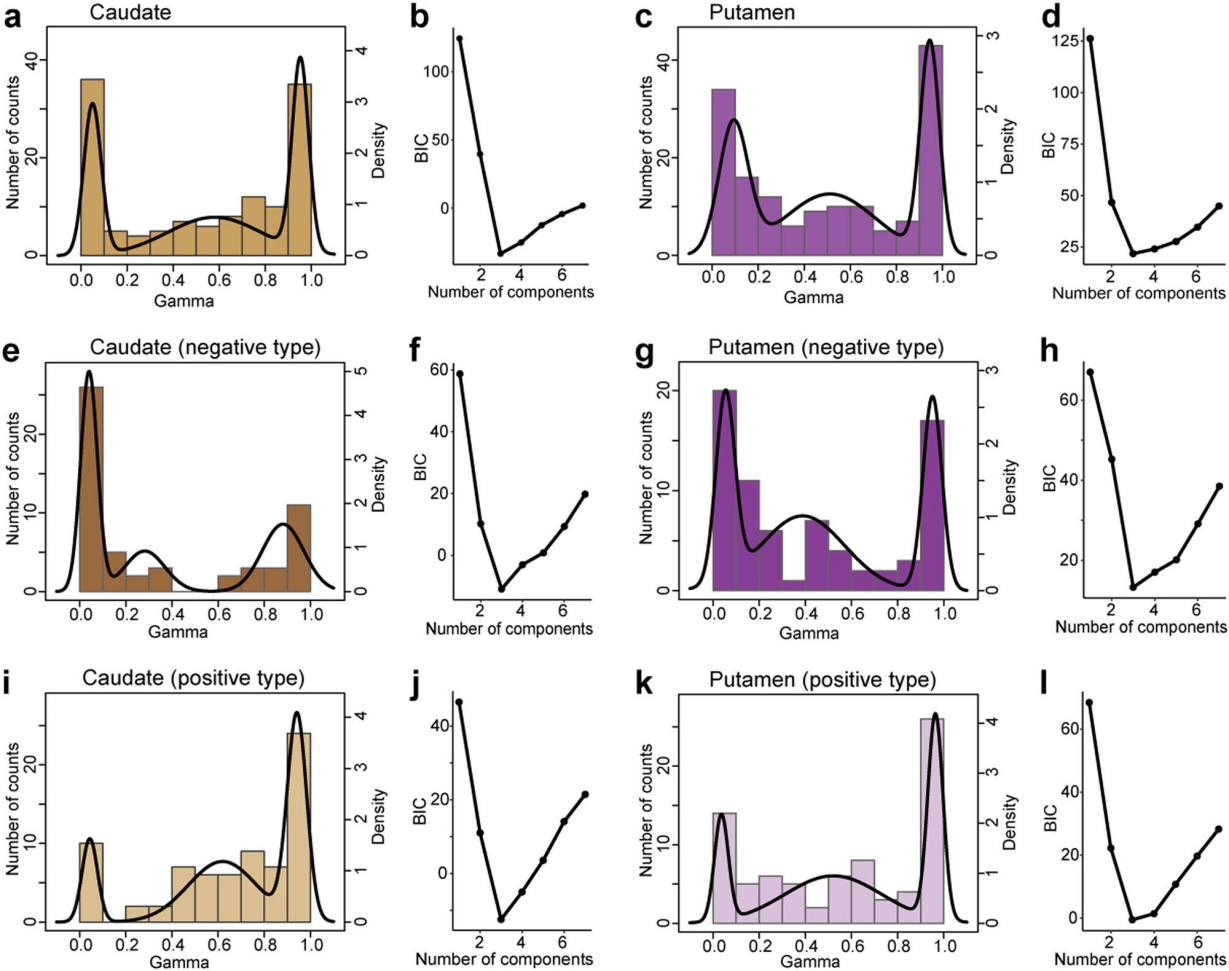
Long-term value signals encoded by PANs are composed of three subgroups. **(a)** Distribution of *γ* values for PANs in the caudate nucleus (bar graph). The best-fitting model is indicated by the black line. **b)** Plots of the estimated Bayesian information criterion (BIC) for the data in **(a)** for each model, which included one to seven components. **(c,d)** Same as **(a,b)** but for PANs in the putamen. **(e,f)** Same as **(a,b)** but for negative-coding type PANs in the caudate nucleus. **(g,h)** Same as **(a,b)** but for negative-coding type PANs in the putamen. **i,j)** Same as **(a,b)** but for positive-coding type PANs in the caudate nucleus. **(k,l)** Same as **(a,b)** but for positive-coding type PANs in the putamen.

In the striatum, PANs in both the caudate nucleus and putamen were composed of three subgroups (Fig. 7a–d) that included large (caudate, mean ± SD: 0.95 ± 0.036, n = 45 activities; putamen, mean ± SD: 0.95 ± 0.042, n = 50), small (caudate, mean ± SD: 0.050 ± 0.038, n = 41; putamen, mean ± SD: 0.093 ± 0.064, n = 50), and intermediate *γ* values (caudate, mean ± SD: 0.58 ± 0.20, n = 42; putamen, mean ± SD: 0.51 ± 0.20, n = 52). In more detail, positive- and negative-coding type PANs in both the caudate and putamen were also consistently composed of three subgroups (Fig. 7e–l) with the consistent *γ* bias as seen in Fig. 5. However, the BIC values were not largely different among the three-, four-, or five-component models in the striatum (Fig. 7b,d), in contrast to the values observed for DA neurons (Fig. 6b,d). This implied that the activity of PANs represented a more heterogeneous timescale for encoding long-term values as compared to DA neurons. These results suggest that DA neurons and PANs do not consist of same subgroups that encode long-term reward values on various timescales.

## Discussion

In the present study, we compared topographic distinctions in encoding long-term values by DA neurons in the midbrain and PANs in the dorsal striatum toward understanding the role of nigrostriatal DA projections. Our findings indicated that DA neurons in the ventromedial part of the midbrain encode values of multiple future rewards on a longer timescale than those in the dorsolateral region. In the dorsal striatum, PANs encoded values of multiple future rewards on both long and short timescales; positive-coding type PANs signaled values reflecting future rewards on a longer timescale than those of the negative-coding type. This segregation of discount factors between positive- and negative-coding PANs was predominantly found in the caudate nucleus, but not in the putamen, both of which receive DA signals from the SNc. The analysis using a mixture of Gaussian distribution models demonstrated that dorsolateral and ventromedial DA neurons are composed of two subgroups in terms of the timescale to encode the values of future rewards, whereas PANs are composed of three or more subgroups. These results suggested that the long-term value signals encoded by presumed striatal output neurons do not closely resemble the DA signals, potentially due to the influence of nigrostriatal projections and inputs from other networks.

Although previous studies have suggested that DA neurons in the midbrain exhibit functional heterogeneity, none have reported clear differences with regard to long-term value coding. Using a standard reinforcement learning model, the present study demonstrated that ventromedial DA neurons represent long-term reward values on longer timescales than dorsolateral neurons (Fig. 4). These observations are consistent with the topographic differences observed in previous studies, in which dorsolateral and ventromedial DA neurons exhibit distinctive characteristics of encoding values^12,13^. For instance, Matsumoto *et al*. reported that ventromedial DA neurons signal the values of rewarding and aversive events as predicted based on the reinforcement learning model, whereas a subset of dorsolateral DA neurons do not do so, instead signaling rewarding and aversive events to reflect saliency^12^. Our findings in ventromedial DA neurons appeared to be consistent with this study since the ventromedial DA neurons in their study and ours might simply reflect the predicted values of single and multiple rewards, respectively. In contrast, comparison of the activity in dorsolateral DA neurons seemed to be difficult because we did not use an aversive stimulus as in their study. For another example, functional magnetic resonance imaging studies in humans have demonstrated that distinct clusters of midbrain regions are preferentially activated by either reward or novel stimuli with distinction between mediolateral and rostro-caudal areas^32^. These previous findings support our conclusion that the dorsolateral and ventromedial DA neurons exhibited distinctive patterns of encoding long-term values during decision making.

Anatomical literature suggests that DA neurons exhibit spatially heterogeneous afferent and efferent projections to and from other brain regions^11,33^. In the present study, dorsolateral midbrain DA neurons represented reward values with small *γ* values. Ventromedial midbrain DA neurons, which may have included recordings from the ventral tegmental area (VTA) which receive inputs from the limbic system, including the ventral striatum, represented reward values with large *γ* values (Fig. 4). It is likely that the observed differences between dorsolateral and ventromedial DA neurons are due to the presence of heterogeneous connections with the target structures (Fig. 1c); the dorsolateral part of the SNc mainly projects to the dorsal part of the caudate nucleus and putamen, the ventromedial part of the SNc projects to the central part of the caudate nucleus and putamen as well as the dorsal part of striatum, and the ventromedial part of the SNc and VTA projects to the ventral striatum^11,16^. Indeed, one previous study is consistent with this anatomical literature based on measurements of blood oxygen level-dependent activity in the human striatum, as the observed ventroanterior-to-dorsoposterior gradient was associated with increased *γ* values^34^. Note however that the topography observed for *γ* values was not consistent with that observed for DA neurons in our study.

A recent anatomical study that utilized a cell-type specific trans-synaptic tracing technique has suggested a different anatomical model for DA neurons. In this model, most DA neurons receive a similar set of inputs rather than reciprocal connections with target brain regions, except those projecting to the tail of the striatum^35^. This anatomical finding is supported by another recent study^36^, in which the tail-projecting dopamine neurons, localized in the caudal-lateral part of SNc, stably retained past-learned reward values of visual objects, while other types of dopamine neurons, localized in the rostral-medial part of SNc, quickly changed their value-related activity through learning. These two types of DA activity could be explained by the reinforcement learning theory in terms of learning rate *α*. The tail-projecting dopamine neurons may reflect low learning rate, while the other dopamine neurons may reflect high learning rate. Although a direct comparison between their study and ours would not be possible, we did not find a significant relationship between learning rate and recording depth (Supplementary Fig. 4a).

Why is the relationship between the *γ* value and recording depth in DA neurons not very strong? One possible reason is that estimated parameters in each single neuron may contain a certain level of noise because single neuron activity has trial-by-trial variability. Heterogeneity of neuronal activity, which occurs outside of the model assumption, also increases the noise in estimating parameters. Thus, the weak but significant topographic relationship with estimated parameters can help in understanding the role of nigrostriatal dopamine projections.

The striatum is thought to integrate sensory and motor information for learning and action execution as it exhibits distinct loop circuits with many cortical areas, including the prefrontal, medial frontal, cingulate, and premotor and primary motor cortices^37^. It also exhibits strong loop connections with the SNc^11^. In our dataset where we recorded from the whole dorsal striatum, we observed no significant differences in long-term value signals, with the exception of those in the positive- and negative-coding types between the caudate nucleus and putamen (Fig. 5). This difference cannot be predicted from the nigrostriatal projection. Moreover, DA neurons and PANs exhibited differences in terms of the number of subgroups signaling long-term values (Figs. 6 and 7). These results suggest that topographic organization of long-term value signals in the midbrain is not simply reflected by the activity of presumed projection neurons in the striatum.

**Figure 8:**
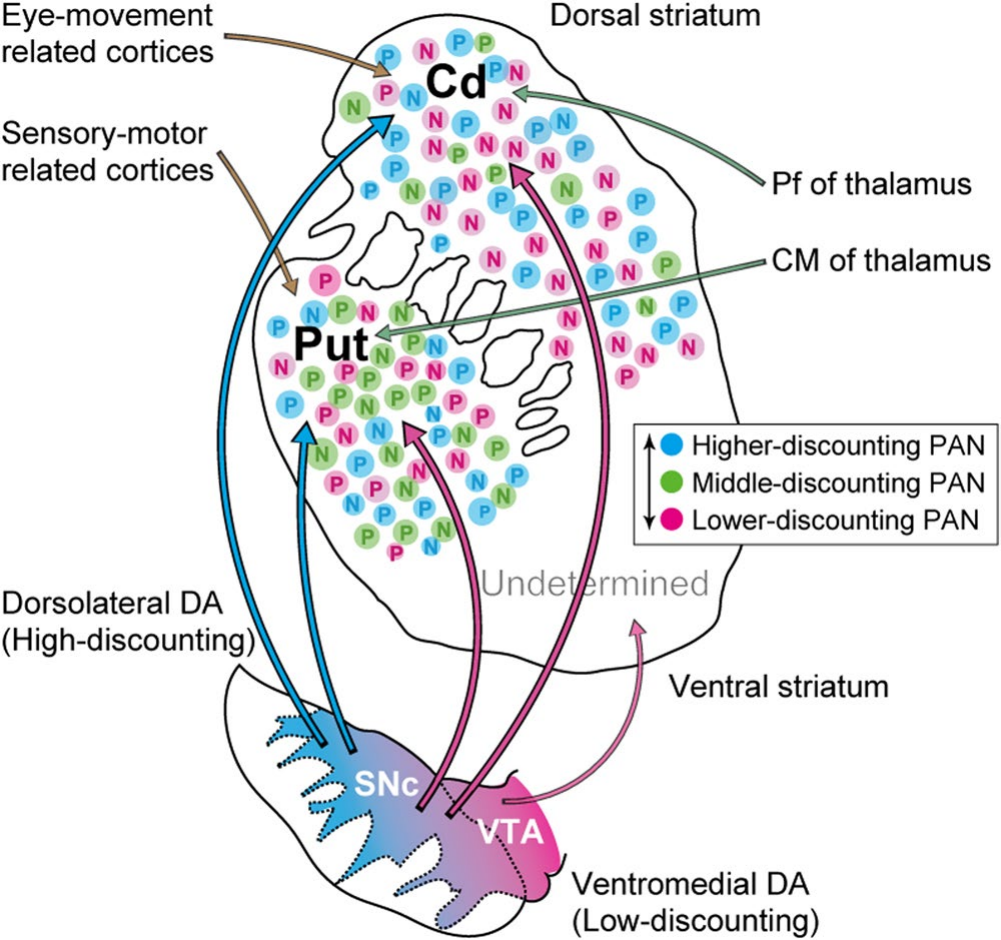
Schematic drawing of the nigrostriatal projection as well as the topography of long-term reward value coding in the midbrain and striatum. Anatomical projections from the midbrain to the striatum based on Haber *et al*., 2000 and the discount factor observed in the present study are shown. PANs representing long-term values with high (cyan), medium (purple), and low (magenta) discounting of future rewards are shown by dots. DA neurons representing long-term values with dorsolateral-ventromedial arrangement are shown by filled colors. Discount factor in the ventral striatum was not examined. Centromedian (CM) and parafascicular (Pf) nuclei of the thalamus; Cd, caudate nucleus; Put, putamen; VTA, ventral tegmental area.

Many previous studies have shown that PANs in the dorsal striatum encode reward values with either a positive or negative regression coefficient^24–27^. Our results were consistent with these previous studies, although our findings were specific to long-term values (Fig. 5a). One possible reason for these contrasting types is differences in the dopamine receptor subtypes, D1- and D2-like receptors^38–40^. Recent studies have shown that the two pathways expressing two distinct subtypes of DA receptors play opposing roles in reinforcement learning and movement control^41–44^, as well as in encoding reward values and outcomes^45–47^. We also found that segregation of discount factors between positive- and negative-coding type PANs was predominantly seen in the caudate nucleus, but was weak and non-significant in the putamen (Fig. 5b). This topographic difference was not predicted from the nigrostriatal projection. It is unlikely that this difference was due to differences in DA receptor subtypes because both D1- and D2-like receptors are similarly expressed in the caudate nucleus and putamen^40^. Such differences may instead be due to the presence of corticostriatal and thalamostriatal projections in the dorsal striatum (Fig. 8). The caudate nucleus mainly receives inputs from the oculomotor cortical areas, whereas the posterior part of the putamen receives inputs from somatosensory and skeletomotor cortical areas^37^. Regarding thalamostriatal projections, the centromedian parafascicular (CM/Pf) complex in the intralaminar nuclei of the thalamus projects to the striatum differentially; Pf neurons mainly project to the caudate nucleus, whereas CM neurons mainly project to the putamen^48–52^. It remains unknown whether differences in long-term value coding are present between these thalamic nuclei, but the neuronal activity in these nuclei in behaving monkeys show contrasting activity patterns^48,53^. Thus, either or both the cortical and thalamic inputs may contribute to the observed difference in long-term value signals among positive- and negative-coding types in the striatum.

In the present study, we revealed that DA neurons are composed of two distinct subgroups signaling the long-term value of future rewards, exhibiting gradual changes along a dorsolateral-ventromedial axis (Figs. 4a and 6). In contrast, PANs are composed of three or maybe more subgroups (Fig. 7). What accounts for this difference, and what does this difference mean? It is unlikely that the procedure used to fit the distribution model yielded this difference, since changes in the quality of fit were clearly different between DA neurons and PANs (Fig. 6b,d vs. Fig. 7b,d). As mentioned above, the dorsal striatum receives DA inputs from both dorsolateral and ventromedial part of the SNc (Fig. 8), from where most of our recordings were made. Thus, PANs in both the caudate nucleus and putamen can be assumed to be under the influence of both dorsolateral and ventromedial DA signals. If we assume that all DA neurons in our dataset project to the whole dorsal striatum and are mixed into one group without distinction of the dorsolateral-ventromedial topography, the presence of three subgroups can best explain the *γ* distributions of DA neurons (Supplementary Fig. 6), as observed for PANs (Fig. 7a–d).

How are these distinctive networks involved in organizing behavioral actions to earn multiple future rewards? One possibility is that the dorsolateral nigrostriatal pathways play a role in motor control along fine and short timescales, while the ventromedial nigrostriatal pathways for cognitive processes involve coarse and long timescales^54^. In accordance with this hypothesis, dorsolateral DA neurons with small *γ* values may reflect processes for motor control in each individual trial, while ventromedial DA neurons with large *γ* values may reflect cognitive processes to achieve far future rewards. Indeed, muscimol-induced inactivation in the posterior part of the dorsal striatum elicits motor deficits (slow movement), while inactivation in the middle part of the dorsal striatum elicits deficits in choice behavior during the multi-step choice task^55^. However, this is unlikely with regard to the representation of long-term values in the dorsal striatum as we observed no significant differences in the estimated *γ* values for AP level, inconsistent with the results of the inactivation study.

Our findings suggest that DA signals along the dorsolateral-ventromedial axis affect the timescale for expected rewards by modulating neuronal activity in the dorsal striatum. Further studies should examine the representation of future rewards in the ventral striatum, especially the nucleus accumbens, and in other prefrontal cortices.

## Materials and Methods

All details regarding analyses of monkey behavior and activity of the DA neurons and PANs have been documented previously^5,31^. New analyses included those of the recording locations and distribution of *γ* values for DA neurons and PANs. All other procedures were identical to those utilized in the two previous studies.

### Subjects and surgical procedures

Four Japanese macaque monkeys were used (*Macaca fuscata*; monkey SK, female, 8.1 kg; monkey CC, female, 7.5 kg; monkey RO, male, 9.4 kg; monkey TN, female, 6.3 kg). Head-restraining bolts and stainless-steel recording chambers were implanted in their skulls in accordance with standard surgical procedures. Monkeys were anesthetized with ketamine hydrochloride (6 mg/kg; i.m.) and pentobarbital sodium (Nembutal, 27.5 mg/kg; i.p.). Recording chambers were laterally positioned under stereotaxic guidance at an angle of 45°. All surgical and experimental procedures were approved by the Animal Care and Use Committee of Kyoto Prefectural University of Medicine and performed in accordance with the National Institutes of Health Guide for the Care and Use of Laboratory Animals in USA.

### Multi-step choice task

The monkeys performed a choice task to obtain multiple rewards through a series of choices^5,31^ (Fig. 1). Briefly, they first searched for a rewarding target among three alternatives in a trial-and-error manner based on the no-reward outcomes in the first (N1), second (N2), or third (N3) trial. After finding a rewarding target, they obtained additional rewards in subsequent (R1 and R2) trials by choosing the rewarded target. Note that in each trial, monkeys made a choice of target, followed by the next trail after inter trial interval (ITI). Monkeys obtained the rewards twice (RO) or three times (SK, CC, and TN) through a series of choices.

In a single trial during the multi-step choice task, the monkeys pressed an illuminated start button (start cue) using the hand contralateral to the side of neuronal recording. Thereafter, three target buttons and a go-LED were simultaneously illuminated. After the go-LED was turned off, the monkeys released the start button and pressed one of the three illuminated target buttons. If they chose a rewarding target button, a drop of a fluid reward was given following a high-tone beep (reward beep). If they chose a non-rewarding target button, no reward was given following a low-tone beep (no-reward beep). The location of the rewarding target button was defined by a computer to adjust the reward rate in N1 trials, which was approximately 20% in monkeys SK and CC and 33% in monkeys RO and TN.

### Analysis of behavioral data

No new behavioral analyses were performed in the present study. All behavioral results during the multi-step choice task have been documented previously. Briefly, mean anticipatory licking durations before the occurrence of outcome beeps, reaction time to the start cue illumination (TST, task start time), reaction time to choose the target buttons (GORT, go reaction time), and movement time from the release of the start cue to pressing of a target button (MT, movement time) were analyzed as per our previous studies. A summary of the behavioral results is described in the Results section.

### Recording of single neuron activity in the midbrain and striatum

We mostly recorded from midbrain DA neurons located around the SNc, but some were from the VTA. DA neurons were identified based on their low tonic spontaneous firing rates (mean ± SD: 4.0 ± 1.4 spikes/s), relatively long duration of action potentials (> 1.5 ms, mean ± SD: 2.2 ± 0.3 ms), transient responses to unexpected reward delivery, and histological verification (Supplementary Fig. 2a), in accordance with previously described methods^56–58^.

We utilized previously identified significant responses for DA neurons. We regarded the activity of DA neurons as a significant response if the firing rates after either the task start cue or outcome beeps increased or decreased significantly from the baseline, estimated during a 500–750 ms baseline window (25 bin) prior to illumination of the start cue. A 75 ms test window was shifted in 10 ms bins up to 450 ms starting from the onset of an event. Significant responses were detected if more than three consecutive comparisons between the test and baseline windows were significantly different (two-tailed Wilcoxon two-sample test, threshold at *p* < 0.05). Onset and disappearance of the response were defined as the beginning and end of consecutive test windows exhibiting statistical significance, respectively. We set quantification windows for the magnitude of DA neuronal activity one SD wider than the windows determined by the average onset and disappearance times of significant changes in firing rate: 40–240 ms after the start cue, 100–340 ms after the reward beep, 80–440 ms after the no-reward beep for monkey SK; 50–290 ms after the start cue, 40–350 ms after the reward beep, 80–410 ms after the no-reward beep for monkey CC.

Although we also utilized PANs identified in a previous study, the detailed procedure for identifying PANs in the dorsal striatum (i.e. caudate nucleus and putamen) was as follows^31^. We differentiated PANs from presumed parvalbumin-containing GABAergic interneurons (FNSs, fast-spiking neurons) and presumed cholinergic interneurons (TANs, tonically active neurons) based on their low spontaneous firing rates (<2 spikes/s) and phasic firings in relation to one or more task events^26^. FSNs and TANs^59,60^ were not analyzed in this study.

We utilized previously identified significant responses of PANs. We estimated the average firing rates during each of the five task periods (start period, 1000 ms preceding and 300 ms following depression of the start cue; pre-Go period, 600 ms preceding the Go signal; target choice period, 300 ms preceding and following depression of the target button; pre-feedback period, 600 ms preceding the outcome beeps; post-feedback period, 2000 ms following the outcome beeps). A significant increase in the firing rate of each of the five task periods was determined by comparing the firing rate during a 150 ms test window with the baseline firing rate for 750 ms prior to illumination of the start cue (two-tailed Wilcoxon two-sample test, threshold at *p* < 0.05). Onset and disappearance of the response were defined as the beginning and end of consecutive test windows exhibiting statistical significance, respectively.

### Estimation of long-term values using a reinforcement learning model

To assess the long-term values for multiple future rewards, we used a standard temporal difference (TD) learning model^61^, same as those utilized in previous studies^5,31^. In this model, the value function, *V*(*S*_*t*_), represents the sum of expected future rewards (*r*_*t*_) discounted by the number of steps to obtain them, starting at state *S*_t_**:**

1 $$V({S}_{t})=E\{(\mathop{\sum }\limits_{k=0}^{\infty }{r}_{t+k+1}|{S}_{t}=S)\}$$

where *E* represents the expectation taken over all states and *k* is an index for future steps. In the multi-step choice task, the state *S*_*t*_ takes values N1, N2, N3, R1, or R2, with R2 as the terminal state. The discount factor, *γ* (0 ≤ *γ* ≤ 1), controls how far rewards are taken into the estimate of the value function. The TD model updates *V*(*S*_*t*_) as follows in proportion to the TD error *δ*_*t*_:

2 $$V({S}_{t})\leftarrow V({S}_{t})+\alpha {\delta }_{t}$$

where *α* is the learning rate (0 ≤ *α* ≤ 1), and *δ*_*t*_ is defined as follows:

3 $${r}_{t}+\gamma V({S}_{t+1})-V({S}_{t})$$

where the first and second terms represent the estimations of *V*(*S*_*t*_) after receiving a fluid reward in milliliters at time *t*. The third term is the same estimation as before receipt of the reward.

### Estimation of γ values in DA neurons and PANs

The procedure used in our previous studies was utilized to fit the reinforcement learning model to the response of DA neurons and PANs^5,31^. The value function *V(S*_*t*_) contains two free parameters: learning rate (*α*) and discount factor (*γ*). The *γ* value was used as a free parameter to explain the activity of neurons. The *α* value was regarded as a constant parameter, as the learning rate becomes stable after substantial training in a static environment. The *α* value was set at 0.02 and 0.2 for DA neurons and PANs, respectively, though different settings of *α* from 0.01 to 1.0 were shown to affect the results only slightly^5^. The *γ* value was used as a free parameter in the simulation in order to represent gradual changes of the estimated *V(S*_*t*_), which exhibited an inverse-V-shaped pattern with medium to large *γ* values (Supplementary Fig. 1a). We ran the TD algorithm to learn the value function during the multi-step choice task, and the value of *V(S*_*t*_) was extracted after 250 (DA neurons, Enomoto *et al*., 2011) or 500 (PANs, Yamada *et al*., 2013) trials/steps. Note that if *V(S*_*t*_*)* was extracted after 250 trials/steps in PANs, the results remained unchanged.

To estimate the best-fit *γ* value for the activity of DA neurons, we first constructed a five-dimensional vector consisting of the mean firing rate of a DA neuron following illumination of the start cue in each state (N1, N2, N3, R1, and R2). We then searched for a *γ* value that maximized the correlation coefficient between the five-dimensional vector consisting of the mean firing rate of a DA neuron and simulated *V(S*_*t*_*)* value.

To estimate the best-fit *γ* value for the activity of PANs, we used a slightly different method, since the activity of PANs was modulated not only by reward values but also by behavioral parameters such as a chosen target, reaction time, movement time, and so on. All possible variables that could explain the neuronal firing rates were included in the model. Neuronal firing rates (*F*) were fitted according to the following model:

4 $$F={b}_{0}+{b}_{1}V({\rm{St}})+{b}_{f}Feedback+{b}_{t}Target+{b}_{TST}TST+{b}_{RT}GORT+{b}_{MT}MT+error$$

where *b*_0_ and error represent the intercept and residual, respectively. *V(S*_*t*_) contains *γ* as a free parameter, as for the fitting in DA neurons. *Feedback* took scalar values in the reward (1) and no-reward (0) trials. The *Feedback* term was included only during the post-feedback period. *Target* took scalar values (1, 0, −1) for the three target options, which were assigned depending on the average firing rates of each target. TST, GORT, and MT were also included in the model to detect the effects of behavioral parameters. We selected the one combination of variables (or model) as well as the estimate of *γ* value that provided the lowest BIC^62^ among all possible combinations of models. Note that nearly identical results were obtained using a simple model that included only *V(St)* and *Feedback* for estimating the best-fit *γ* value in each PAN. We searched for the best-fit *γ* values within the range of 0 to 1.0 in the present study (0.1 to 0.9 was used for PANs in Yamada *et al*., 2013, but 0 to 1.0 was used for DA neurons in Enomoto *et al*., 2011) because the range of *γ* should be identical among DA neurons and PANs for comparisons.

### Histological reconstruction of recorded neurons in the midbrain and striatum

After completing all neuronal recordings, we made small electrolytic lesions along selected electrode tracts in the SNc, VTA, caudate nucleus, and putamen by passing a direct anodal current (20 μA) through tungsten microelectrodes for 30 s. Following perfusion, coronal sections (thickness: 50 μm) were stained with cresyl violet (Nissl stain) and reconstructions were created based on the observed electrode tracts and electrolytic microlesions.

The dorsolateral and the ventromedial DA neurons were defined using an anatomical criterion, which is the midpoint of the SNc between its dorsolateral and ventromedial edges. The recording depths of the dorsolateral and ventromedial edge in the SNc were 27.4 mm and 33.9 mm, respectively. The midpoint between the dorsolateral and ventromedial edges of the SNc was at 30.65 mm. Following this criterion, we defined the dorsolateral and ventromedial dopamine neurons. The PANs from the caudate nucleus and putamen were defined using another anatomical criterion, the edge of the caudate (i.e. mediolateral axis is -1).

### Statistical analysis of the recording location for DA neurons and PANs

We analyzed differences in *γ* values (Figs. 4b and 5) via the Kolmogorov-Smirnov test (*p* < 0.05) and linear regression analyses using MATLAB or R software. In the linear regression analyses, the estimated *γ* value (*Y*) in DA neurons was fitted according to the following model:

5 $$\begin{array}{c}Y={b}_{0}+{b}_{1}DV+{\rm{error}}\end{array}$$

where *b*_0_ and error represent the intercept and residual, respectively. *DV* represents the recording depth from the cortical surface along the dorsolateral-ventromedial axis.

The estimated *γ* value (*Y*) in PANs in either caudate nucleus or putamen was fitted according to the following model:

6 $$\begin{array}{c}Y={b}_{0}+{b}_{1}AP+{b}_{2}DV+{\rm{error}}\end{array}$$

where *b*_0_ and error represent the intercept and residual, respectively. *AP* and *DV* indicate the recording locations of PANs (mm) along the anterior-posterior and dorsolateral-ventromedial axes, respectively (Fig. 2c,d and Supplementary Fig. 2b).

To further examine whether the estimated *γ* value (*Y*) in PANs was dependent on the mediolateral axis in each of the caudate nucleus and putamen, we fitted the following model:

7 $$\begin{array}{c}Y={b}_{0}+{b}_{1}ML+{b}_{2}Coding\,type+{\rm{error}}\end{array}$$

where *b*_0_ and error represent the intercept and residual, respectively. ML indicates the recording locations of PANs (mm) along the mediolateral axis. The *Codingtype* took a scalar value of 0 and 1 for the negative and positive coding type PANs, respectively.

### Gaussian mixture model for the distribution of *γ* values in DA neurons and PANs

A Gaussian mixture model is an approach for identifying subgroups which construct populations of neurons^63,64^. To evaluate the heterogeneity of the DA and PANs, we fitted Gaussian mixture models (GMMs) to the distribution of *γ* values, which determine the number of GMM components that best explain the distribution for DA neurons and PANs using the R software package ‘mclust’. The package provided the maximum likelihood of the model via the expected maximization algorithm^65^, which determines the parameters of the mixture components. We fitted the GMM with variable variances. To ensure Gaussian distribution of the data irrespective of the estimation accuracy and error structure of the estimated *γ* value in the reinforcement learning model, we randomly added very small Gaussian noise (SD=0.05) to the estimated *γ* value before fitting the GMM. We determined the best model to be the one with the lowest BIC value.

### Statistical analysis

Statistical tests were performed using MATLAB and R software. A p value <0.05 was considered to be statistically significant. For Kolmogorov-Smirnov tests, we showed the KS statistic, i.e. the maximum absolute difference between cumulative distributions, as a measure of effect size.

## Supplementary information

Supplementary information.

## Data Availability

The datasets in the current study are available from the corresponding author on reasonable request.
