# Supplementary material for: Topographic distinction in long-term value signals between presumed dopamine neurons and presumed striatal projection neurons in behaving monkeys
Source: Sci Rep. 2020 Jun 2;10:8912. doi: 10.1038/s41598-020-65914-0 (PMC7265398; doi:10.1038/s41598-020-65914-0)
Supplement: Supplementary file 1 — Supplementary information. [file 41598_2020_65914_MOESM1_ESM.docx]

**Topographic distinction in long-term value signals between presumed dopamine neurons and presumed striatal projection neurons in behaving monkeys**

Kazuki Enomoto, Naoyuki Matsumoto, Hitoshi Inokawa, Minoru Kimura, Hiroshi Yamada

**Supplementary Results:**

**Ventromedial DA neurons exhibited larger responses than dorsolateral neurons after the start cue and outcome beeps**

In order to verify the modulation of long-term value signals according to depth, we also examined the magnitude of DA responses without the reinforcement learning model. We applied a linear regression analysis to the activity of all DA neurons in each trial type (Supplementary Fig. 3a). Across our population of DA neurons, consistent changes in response magnitudes were observed in accordance with the recording depth. The deeper ventromedial DA neurons exhibited greater responses to the start cue than the shallower dorsolateral DA neurons, as indicated by the positive regression slopes to the depth, especially in N2 and N3 trials (Supplementary Fig. 3a, left, general linear model, trial type, *p* < 0.0001, F(4,250) = 6.755; recording depth, *p* < 0.0001, R^2^ = 0.154). Depth-dependent changes in activity were consistently observed with and without statistical significance when outcome beeps appeared (reward beep, trial type, *p* < 0.0001, recording depth, *p* < 0.0001, R^2^ = 0.551; no-reward beep, trial type, *p* = 0.542, recording depth, *p* < 0.0001, R^2^ = 0.321), during which reward prediction error (the difference between the predicted long-term values and outcomes) manifested for reward and no-reward beeps. In contrast, there was no significant regression coefficient between recording depth and baseline activity of DA neurons (Supplementary Fig. 3b, linear regression, *r* = 0.06, *p* = 0.707, R^2^ = 0.00292). Thus, ventromedial DA neurons exhibited larger changes in activity than dorsolateral neurons after the start cue and outcome beeps.

**Supplementary Materials and Methods**

To examine whether the firing rates of DA neurons (*F*) were dependent on the recording depth and trial type, we fitted the following model:

$$\begin{aligned} F=b_{0}+b_{1}DV+b_{2}Trialtype+error \#\left( S1 \right) \end{aligned}$$

where *b_0_* and error represent the intercept and residual, respectively. *DV* represents the recording depth from the cortical surface along the dorsolateral-ventromedial axis. The *Trialtype* is a categorical variable composed of N1, N2, N3, R1 and R2, respectively. The firing rates (*F*) were estimated and used for the analysis during the start cue, reward beep, and no-reward beep, respectively.

To examine whether the baseline firing rates of DA neurons (*F*) were dependent on the recording depth, we fitted the following model:

$$\begin{aligned} F=b_{0}+b_{1}DV+error \#\left( S2 \right) \end{aligned}$$

where *b_0_* and error represent the intercept and residual, respectively. *DV* represents the recording depth from the cortical surface along the dorsolateral-ventromedial axis. The baseline firing rates (*F*) were estimated and used for the analysis during a 500 – 750 ms baseline window prior to illumination of the start cue.


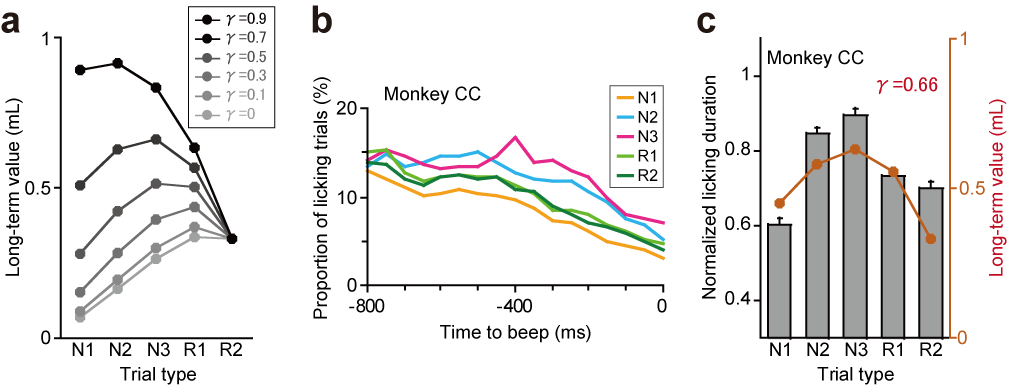
**Supplementary Figures:**

**Supplementary Figure 1. Anticipatory licking behavior reflects the long-term reward value.**

**a)** Long-term values with various *γ* values estimated using the standard reinforcement learning model. **b)** Average proportion of trials in which the amplitude of anticipatory licking exceeded 50% of maximum amplitude plotted against the time to the outcome beep. **c)** Normalized licking duration during the 100 - 800 ms period before outcome beeps in each trial type (bar graph; mean and S.E.). Superimposed line plot indicating the long-term value (right axis) estimated based on the best-fitting *γ* value (0.66). See Enomoto et al., 2011 for more detail.


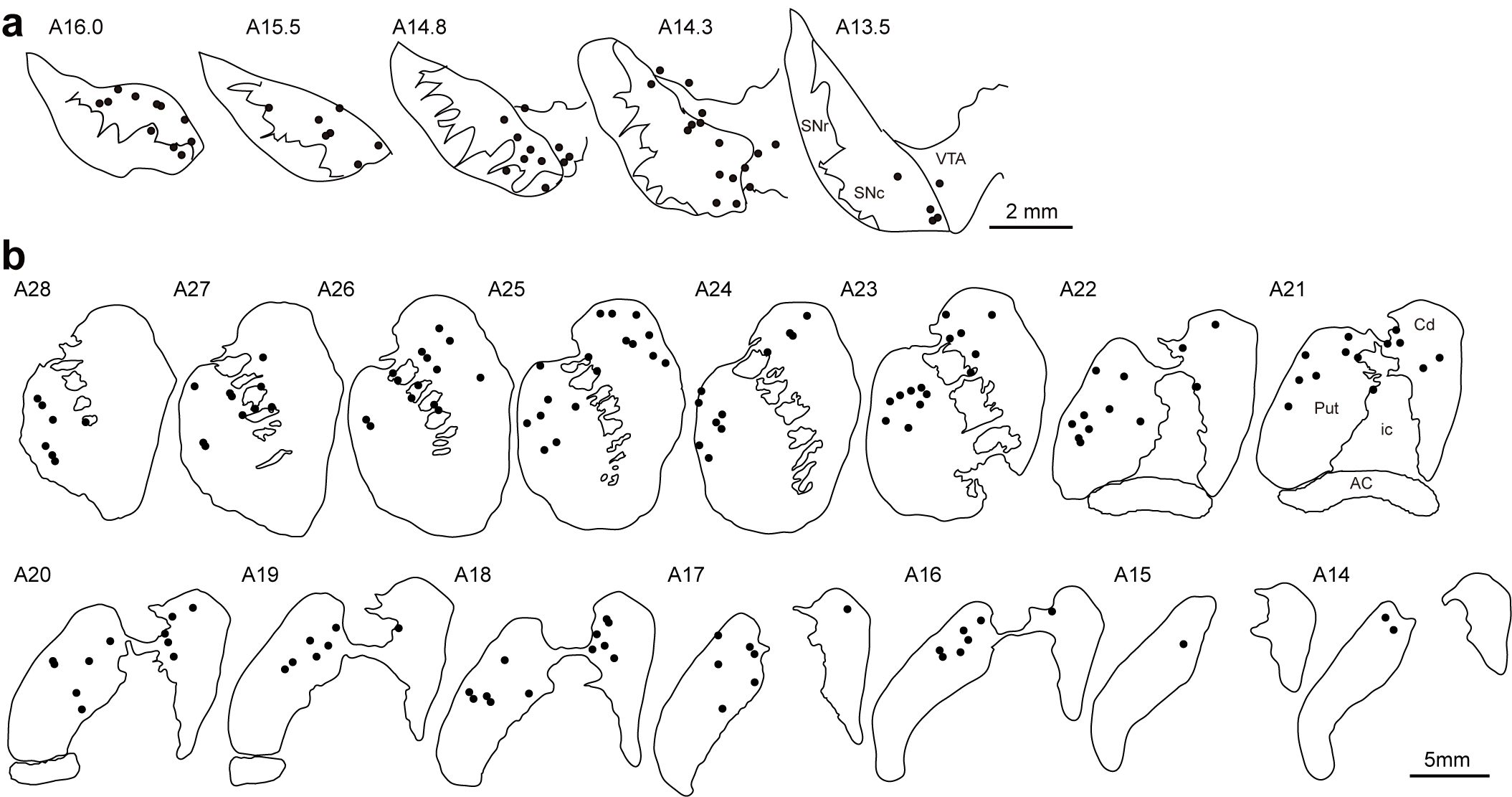
**Supplementary Figure 2.** **Histological reconstruction of recording sites for DA neurons in the midbrain and PANs in the dorsal striatum.**

**a)** Histological reconstruction of DA neurons (black dots) on coronal sections. SNr, substantia nigra pars reticulata; SNc, substantia nigra pars compacta; VTA, ventral tegmental area. **b)** Same as **(a)** for PANs. Cd, caudate nucleus; Put, putamen; ic, internal capsule; AC, anterior commissure.


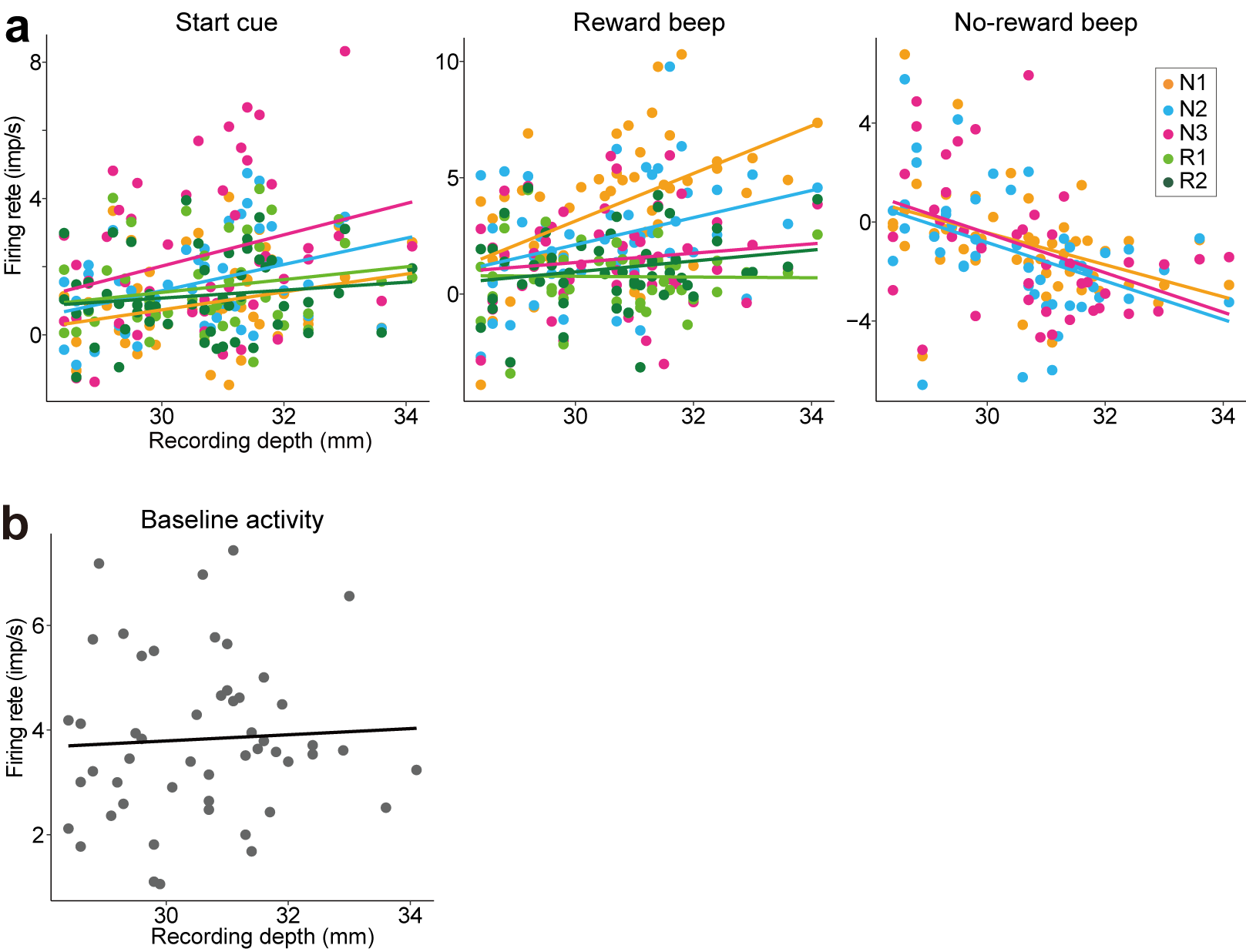
**Supplementary Figure 3. Response amplitudes of DA neurons increase along with recording depth.**

**a)** Response amplitude of all DA neurons, relative to baseline activity, to the start cue (left panel), reward beep (center panel), and no-reward beep (right panel) according to the recording depth. Regression lines in each trial type are differentiated by color (N1, orange; N2, cyan; N3, magenta; R1, light green; R2, dark green). **b)** Same as **(a)** but for the baseline activity of the 51 DA neurons. Gray line indicates the regression line.


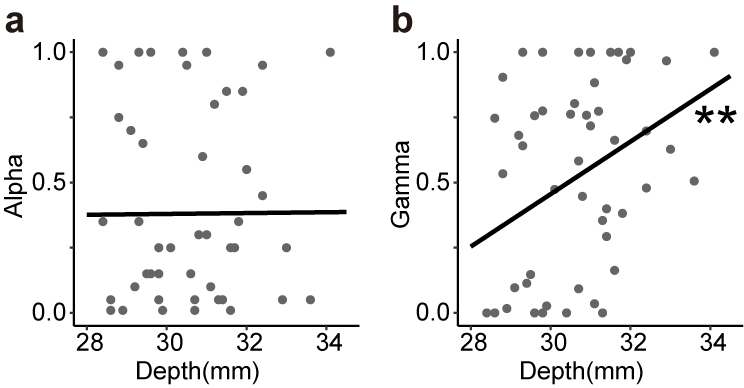
**Supplementary Figure 4. The recording depth of the DA neurons does not depend on the learning rate, but on the discount factor.**

**a)** Scatter plot of the estimated *α* values (learning rate against recording depth of DA neurons. **b)** Same as **(a)** but for the estimated *γ* values. Black lines indicate the regression lines. Asterisks indicate the significance of the regression coefficient (***p* < 0.01).


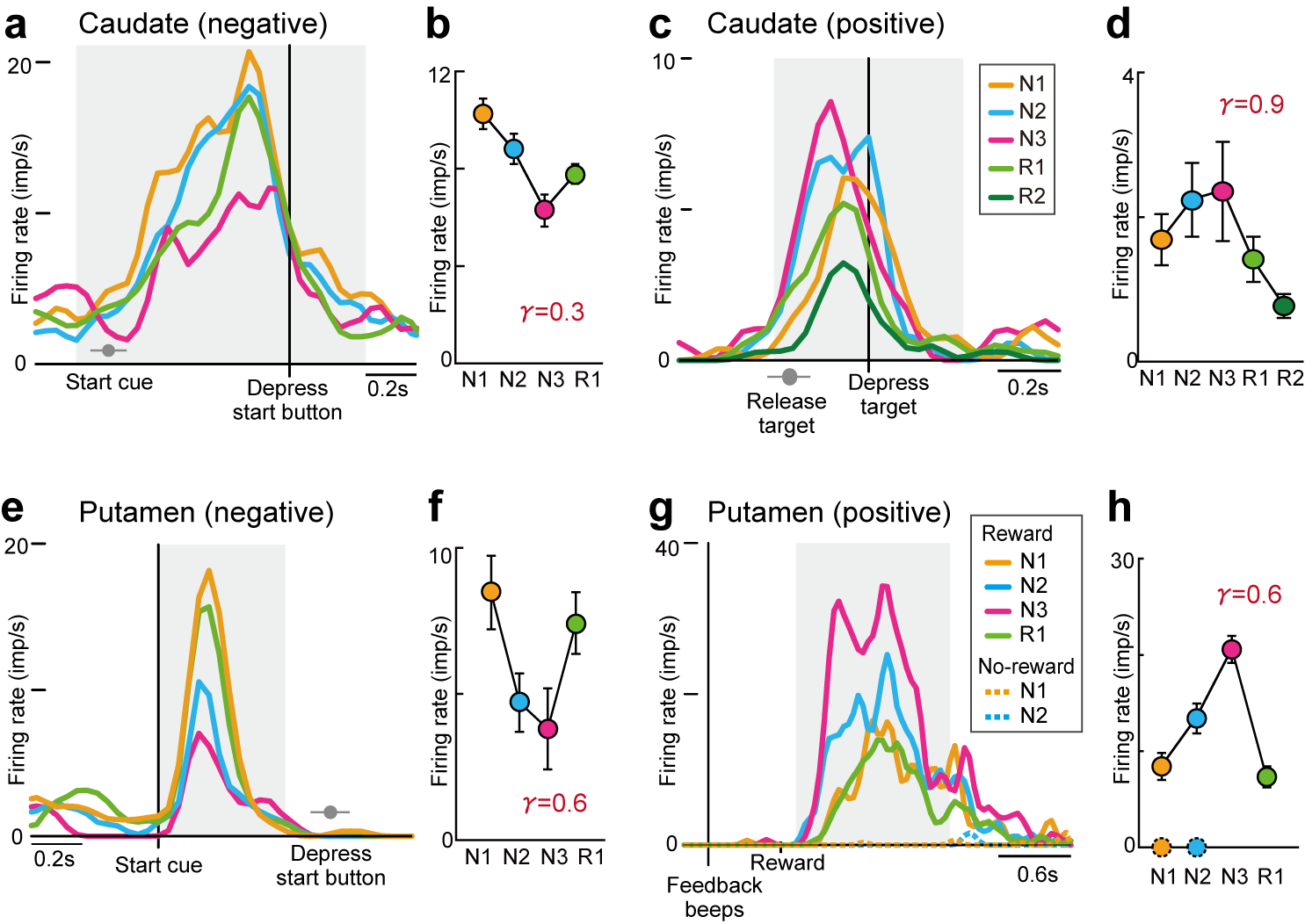
**Supplementary Figure 5. Activity of PANs reflect long-term reward value during various task periods.**

**a)** Representative activity histogram for negative-coding type PANs in the caudate nucleus. Histograms are sorted according to trial type (N1, orange; N2, cyan; N3, magenta; R1, light green; R2, dark green). The vertical line represents depression of the start button. The hatched gray area corresponds to the time window used to measure response amplitude. The gray circle indicates the average latency of the start cue (see Yamada et al., 2013 for details). **b)** The average firing rate in the hatched gray area in **(a)** in each trial type (mean and S.E.). **c, d)** same as **(a, b)** for a positive-coding type PAN in the caudate nucleus before and after target button depression. **e-h)** same as **(a-d)** but for putamen neurons after start cue **(e, f)** and after outcome beeps **(g, h)**.


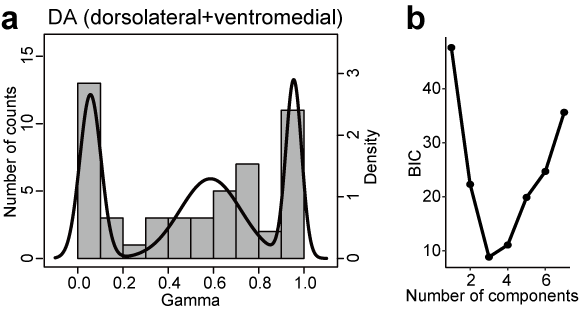
**Supplementary Figure 6. Fitting of the Gaussian mixture models to all DA neurons.**

**a)** Distribution of *γ* values for all DA neurons (bar graph). The best-fitting model is indicated by the black line. **b)** Plots of the estimated Bayesian information criterion (BIC) to the data in **(a)** for each model, which included one to seven components.

**Supplementary Tables:**

**Supplementary Table 1.** **Percentage of rewarded choices in each trial type after training.**

|  | Trial type | | | | |
| --- | --- | --- | --- | --- | --- |
| Monkey | N1 | N2 | N3 | R1 | R2 |
| SK | 17.2±3.4 | 49.1±6.0 | 89.1±6.7 | 97.1±2.2 | 97.0±1.4 |
| CC | 20.1±3.7 | 47.3±4.2 | 76.0±6.5 | 95.9±3.1 | 95.0±2.6 |
| RO | 33.0±3.5 | 50.0±4.5 | 88.7±9.0 | 96.1±4.7 | – |
| TN | 32.3±3.0 | 48.9±4.8 | 81.6±10.8 | 93.2±6.7 | 95.6±6.0 |

Values represent the mean and S.D. of the percentage at which rewarding targets were chosen by monkeys. N1, N2, N3, R1, and R2 indicate the trial types, as shown in Fig. 1b. Percentages to find a rewarding target in N1 trials were set at less than chance (20%) in monkeys SK and CC and at one third of chance level in monkeys RO and TN.

**Supplementary Table 2.** Number of recorded neurons during the multi-step choice task.

|  | DA neurons | | | PANs | | |
| --- | --- | --- | --- | --- | --- | --- |
| Monkey ID | SK | CC | Total | RO | TN | Total |
| Number of cells | 26 | 25 | 51 | 145 | 147 | 292 |

DA: dopamine; PAN: phasically active neurons
